# The preservation of microbial DNA in archived soils of various genetic types

**DOI:** 10.1371/journal.pone.0173901

**Published:** 2017-03-24

**Authors:** Ekaterina A. Ivanova, Ilia O. Korvigo, Boris F. Aparin, Evgenii L. Chirak, Elizaveta V. Pershina, Nikolay S. Romaschenko, Nikolai A. Provorov, Evgeny E. Andronov

**Affiliations:** 1 Laboratory of Microbiological Monitoring and Bioremediation of Soils, All-Russia Research Institute for Agricultural Microbiology, Saint-Petersburg, Russia; 2 Laboratory of Biology and Biochemistry of Soils, V.V. Dokuchaev Soil Science Institute, Moscow, Russia; 3 Laboratory of Functional Analysis of the Genome, Moscow Institute of Physics and Technology, Moscow, Russia; 4 ITMO University, Saint-Petersburg, Russia; 5 Department of Soil Science and Soil Ecology of Saint-Petersburg State University, Saint-Petersburg, Russia; 6 Department of Microbiology and Genetics of Saint-Petersburg State University, Saint-Petersburg, Russia; University of Massachusetts, UNITED STATES

## Abstract

This study is a comparative analysis of samples of archived (stored for over 70–90 years) and modern soils of two different genetic types–chernozem and sod-podzolic soils. We revealed a reduction in biodiversity of archived soils relative to their modern state. Particularly, long-term storage in the museum exerted a greater impact on the microbiomes of sod-podzolic soils, while chernozem samples better preserved the native community. Thus, the persistence of microbial DNA in soil is largely determined by the physico-chemical characteristics that differ across soil types. Chernozems create better conditions for the long-term DNA preservation than sod-podzolic soils. This results in supposedly higher levels of biodiversity conservation in the microbiomes of chernozem with preservation of major microbial taxa dominant in the modern (control) soil samples, which makes archived chernozems a promising object for paleosoil studies.

## Introduction

Paleomicrobiology is a fast-developing area of research in the modern biological science. A large amount of data has been recently accumulated about the ancient microbiomes of different origin: bacterial communities of oral and dental pulps, bones, microbial complexes associated with coprolites [1, 2, 3]. Such studies are possible due to high levels of microbial DNA preservation in the environment: for example, microbial DNA, successfully extracted from permafrost samples, is up to 400,000–600,000 years old [4]. Unfortunately, scholars mostly tend to focus on investigating ancient human-associated microbiomes, while a large portion of microbial genetic information accumulated in the soil environment remains unexplored.

Soil is a large source of biological diversity and genetic information: 1 gram of soil can harbor up to 10 billion of different microorganisms, most of which (up to 90–99%) cannot be cultivated in the lab [5, 6, 7]. This enormous diversity is due to soil’s unique characteristics as a habitat, i.e. the simultaneous presence of three different phases: solid, liquid and gaseous, as well as a high internal surface area. The presence of soil structure–soil aggregates of various sizes—causes the coexistence of numerous microcosms with strikingly different and sometimes opposing conditions. Aggregate structure and the presence of soil organic matter and organo-mineral complexes create favorable conditions for the accumulation and persistence of DNA in the soil environment [8, 9, 10].

Soil formation is a long process, which makes the study of soil dynamics over long time intervals (centuries and millennia) particularly interesting. Such analyses of microbiocenosis of ancient soil samples and sediments can be used to study spatial features of pedogenesis in early geological periods, as well as the dynamics of the natural environment on a geologic time scale. Several papers about fossil soils from different regions around the globe have been published recently, revealing the presence of viable microorganisms. These findings were supported by the studies of permafrost soils of the Arctic and Antarctic and by the studies of deep sedimentary rocks, which were not exposed to cryogenic effects, as well as by the study of buried soils beneath barrows [11, 12, 13].

From this perspective, the preservation of microbial DNA in soils, formed hundreds of years ago and stored in soil museums for prolonged periods of time, is especially interesting. Recent studies have evinced the value of archived soil samples in terms of microbiological information [14]. The comparative analysis of archived soils and their modern counterparts showed a significant increase in the expression of drug resistance genes in arable soils over time, which may be associated with the widespread adoption of antibiotics since 1940 [15]. Other studies have shown viable culturable bacteria isolated from archived soil samples [16, 17]. In these studies the presence of *Bacillus asahii* was detected in samples exposed to manure, while these bacteria were almost completely absent in the soil microbiomes from conventional farming systems [17].

Soil archives–a collection of samples from various regions–exist in many countries [18]. In Russia, the largest collection is deposited in FSBSI «The Central Museum of Soil Science by V.V. Dokuchaev». This museum was established by V.V. Dokuchaev—the founder of genetic soil science in Russia, and is one of the largest museums focusing on soil science and environment. The museum is focused on the accumulation of soil samples from various areas, the systematization of data on soil properties and soil conditions, as well as fundamental and applied (agricultural) research. Currently, the museum's collection includes over 1,600 soil monoliths–vertical sections of different soils of undisturbed structure, and thus, provides a rare and unique opportunity to analyze microbial communities of soils formed over a hundred years ago under different environmental conditions, as well as to investigate the evolutionary dynamics of soil microbiomes within the global soil-forming process.

To carry out such a research it is crucial to have archived samples with recorded sampling sites to sample modern day controls. However, the differences in soil mineralogical composition and particle size as well as the differences in the organic composition are likely to affect the levels of microbial DNA preservation. Thus, this study had two major objectives: first, to analyze the microbial communities of archived samples of contrasting soil types (chernozems and sod-podzolic soils), and second, to compare the data obtained with the microbiome analysis of modern soils formed under similar climatic conditions.

## Materials and methods

### Soil samples

Samples of archived sod-podzolic (ASp) and chernozem (ACh) soils were collected from the top horizon (0–25 cm) of soil cores of the Central Museum of Soil Science by V.V. Dokuchaev. Initial soil cores of sod-podzolic archived soil were sampled from the field near village Lisino (Leningradskaya region, Tosnensky district). No permissions were required for sampling the soils investigated in this study. Russian law doesn't regulate soil sampling, unless it happens in private/classified areas or in national parks, none of which applies here. The study does not involve endangered or protected species.

Soil cores of chernozem were delivered from sampling sites within the Kamennaya Steppe Reserve (Voronezhskaya region, Talovsky district) with different vegetation: fallow grassland, field with continuous winter wheat cropping and forest belt (deciduous forest) (Table 1). Sampled soil cores were immediately air-dried on delivery to the museum until they reached the condition of hygroscopic moisture. After that they were stored in lacquered wooden containers with glass lids in the museum hall at constant temperature and humidity (T 22-25ºC, W 60%). Samples of the top horizon (0–20 cm) of modern soils of the same types were used as a control in the community analyses. Modern soils were extracted from the Soil Collection of All-Russia Research Institute for Agricultural Microbiology. The samples were chosen according to the sampling site of archived soils (or close to that region). Samples of modern sod-podzolic soils (Sp) originated from two places: field near village Lisino (Leningradskaya region, Tosnensky district, N 59°26'08'', E 30°39'52'') and the fallow grassland soil of Leningrad Agricultural Institute (Leningradskaya region, village Belogorka, N 59°20'55.48'', E 30°08'53.53'') (about 40 km from village Lisino). Samples of modern chernozem (Ch) had the same geographic location (Table 1) and were collected in the same land use conditions as their archived counterparts. After collection, samples of modern soils were frozen at -70 degrees Celsius for subsequent DNA extraction.

**Table 1:** Description of the soil samples.

Sample ID	Soil Type (Russian Soil Classification, 2004)	Soil Group (WRB, 2006)	Year of collection	Biome	Sampling area
**ACh1**	Chernozem	Chernozems Chernic	1929	Grassland	Kamennaya Steppe Reserve
**ACh2**			1929		
**ACh3**			1929		
**ACh4**			1929	Field	
**ACh5**			1929		
**ACh6**			1929		
**ACh7**			1947	Forest belt	
**ACh8**			1947		
**ACh9**			1947		
**ASp1**	Sod-podzolic	Umbric Albeluvisol	1934	Field	Leningradskaya region, village Lisino
**ASp2**			1934		
**ASp3**			1934		
**ASp4**			1950		
**ASp5**			1926		
**ASp6**			1949		
**ASp7**			1932		
**Ch1**	Chernozem	Chernozems Chernic	2015	Field	Kamennaya Steppe Reserve
**Ch2**			2015		
**Ch3**			2015		
**Ch4**			2015	Grassland	
**Ch5**			2015		
**Ch6**			2015		
**Ch7**			2015	Forest belt	
**Ch8**			2015		
**Ch9**			2015		
**Sp1**	Sod-podzolic	Umbric Albeluvisols	2014	Field	Leningradskaya region, village Lisino
**Sp2**			2014		
**Sp3**			2014		
**Sp4**			2015	Grassland	Leningradskaya region, village Belogorka
**Sp5**			2015		
**Sp6**			2015		
**Sp7**			2015		
**Sp8**			2015		

### DNA extraction

DNA was extracted from 0.2 g of soil using PowerSoil DNA Isolation Kit (MO BIO, US), which included a bead-beating step, according to the manufacturer’s specifications (MoBio Laboratories, Solana Beach, CA). Homogenization of the samples was performed using Precellys®24 (Bertin Technologies, France). The DNA purity and quantity were tested by electrophoresis in 0.5× TAE buffer on 1% agarose. The average DNA yield was 2–5 μg DNA with the concentration of 10–50 ng/μl. DNA concentrations were additionally estimated using a Qubit 2.0 fluorometer (Thermo Fisher Scientific, USA).

### Quantitative PCR analyses

The abundancies of bacterial small subunit rRNA gene copies were analyzed by qPCR (reaction volume 25 μl) using iQ™ SYBR Green Supermix (BIO RAD). The forward primer Eub338 and reverse primer Eub518 were used [19]. Reference curves were generated using a 10-fold serial dilution of a plasmid containing a full-length copy of an E. coli 16S rRNA. All qPCR reactions were run in triplicate. The reaction was carried out in a CFX96 Touch machine (BIO RAD) using the following protocol: 94°C for 15 min, followed by 40 cycles of 94°C for 30 s, 50°C for 30 s and 72°C for 30 s.

### Amplicon library preparation and bar-coded pyrosequencing of archaeal and bacterial communities

The purified DNA templates were amplified using universal multiplex primers F515 5’-GTGCCAGCMGCCGCGGTAA-3’ and R806 5’-GGACTACVSGGGTATCTAAT-3’ [20] targeting the variable region V4 of bacterial and archaeal 16S rRNA genes. Each multiplex primer contained an adapter, a 4-bp key (TCAG), a 10-bp barcode and the primer sequences. The expected length of the amplification product was 400 bp. Purification, pooling and pyrosequencing of the amplicons were performed with per manufacturer’s instructions (Roche, US). Pyrosequencing was carried out using a GS Junior system (Roche, US).

### Bioinformatics of the pyrosequencing-derived dataset

The raw sequences were processed using QIIME ver. 1.9.1 (www.qiime.org). To reduce sequencing errors, the multiplexed reads were first filtered for quality and grouped by the barcode sequences. Sequences were discarded if they were less than 200 bp long, had a quality score below 25, contained invalid barcodes, primers, ambiguous characters or a homopolymer exceeding 7 bp. All uncategorized ribosomal sequences and chimeras were also removed from the dataset. In total, 49 577 filtered sequences were generated from the archived soil samples with an average of 2490 sequences per library. The minimum, median and maximum lengths of sequences were 200, 355 and 313 bp, respectively. Similar sequences were de-novo clustered into operational taxonomic units (OTUs) with the identity threshold of 97%. A representative set of sequences was chosen by selecting the most abundant sequence from each cluster. Representative sequences were classified using the RDP classifier [21] with a confidence level of 80% and aligned using PyNast [22] and Greengenes database [23]. Aligned sequences were used to build a distance matrix with a distance threshold of 0.1 and a phylogenetic tree necessary for downstream analysis. The sequencing data of archived soils were deposited in the SRA database (accession SRP075197).

To compare microbial communities, alpha- and beta- diversity analyses were performed. To estimate alpha-diversity, we measured richness (in terms of the number of observed species, the Сhao index and the Faith’s phylogenetic diversity) and entropy (via the Shannon-Wiener index). For beta-diversity, the weighted UniFrac metric [24] was used to calculate the amount of dissimilarity (distance) between any pair of bacterial communities. The results were presented in PCoA analysis Emperor plots with jackknife statistics. All estimates were measured for the normalized data (normalization was carried out up to the smallest number of sequences present in the sample).

### Statistical analyses

We analyzed ordination using three distance measures: weighted and unweighted UniFrac, and Bray-Curtis dissimilarity. To compensate for unequal sequencing depth and account for variance, we calculated the distance matrices by averaging pair-wise distances from 100 random subsamples of 900 observations from the OTU table. We first tested the separation between the communities of archived and modern soils using anosim. To further assess whether soil type affects the level of age-related separation we used adonis, which, unlike anosim, can analyze both categorical and continuous factors, as well as factor interactions. We incorporated sample age, sample type (archived or modern) and soil type into the model, as well as the interaction between the latter two. We used anosim and adonis implemented in package *vegan* for the R language.

To assess age-related changes in the community composition we used beta-regression, which naturally models fraction-variables. Since we wanted to account for the vegetation type and the interaction between this variable and the sample type, we could only take a subset of our samples with completely balanced category groups. These included all chernozem specimens from 1929 and 2015 sampled at grasslands and fields.

All statistical analyses were carried out in R. We used package *vegan* for ordination [25] and package *betareg* [26] for beta-regression models.

## Results

### Total DNA extraction and qPCR evaluation

**Fig 1:**
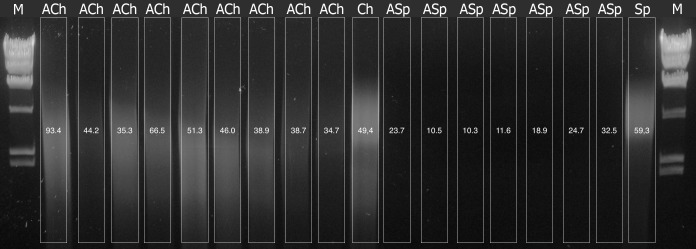
Electropherogram of DNA in agarose gele. М–marker of λ phage DNA treated by Hindt 3 restrictase, ACh–Samples of Archived Chernozem (ACh), Ch–Modern (Control) Chernozem (Ch), ASp–Samples of Archived Sod-podzolic soil (ASp), Sp–Modern (Control) Sod-podzolic (Sp) soil. Numbers show DNA concentration in ng/μl.

The analysis of electropherograms of purified DNA revealed the presence of high-quality DNA in ACh samples whereas the lanes of ASp samples showed no visible trace of DNA (Fig 1). At the same time, ASp DNA concentrations were high enough to produce visible traces, which hinted that most of this DNA was heavily degraded.

**Fig 2:**
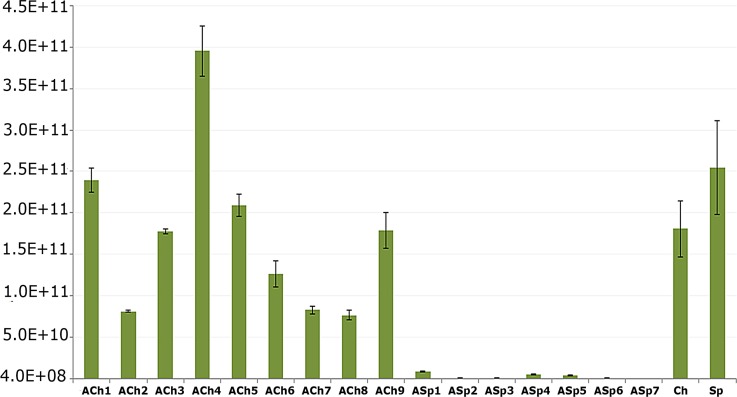
qPCR analysis of 16 S rRNA gene of bacteria in archived and modern soils. ACh1-9 –samples of Archived Chernozem, ASp1-7 –samples of Archived Sod-podzolic soils, Ch–sample of modern Chernozem, Sp–sample of modern Sod-podzolic soil. Data are expressed as mean ± standard deviation. Numbers above the bars show 16S rDNA abundance in 10^8^ copies per gram of soil.

The analysis of qPCR data of 16 rRNA gene showed that the quantity of bacterial 16 S rRNA ribosomal operons per gram of ASp soil was 1.5 order of magnitude less than that for ACh soil (Fig 2). Ch and Sp were similar in quantity of bacterial abundance.

Thus, chernozem environment provides more favorable conditions for preservation of a good-quality DNA, both total and prokaryotic, than sod-podzolic soil, where DNA mostly degraded, according to electrophoresis and qPCR data.

### α-diversity of soil microbial communities

The biodiversity of soil samples was measured in terms of the number of observed species, the Сhao index, the Shannon-Wiener index and phylogenetic diversity (Faith PD). To compare the diversity of archived soils with the modern analogs all the samples of each type (archived sod-podzolic, archived chernozems, modern sod-podzolic and chernozems) were pooled (and normalized to the lowest number of sequences in dataset of each soil type) to estimate the integral differences in their biodiversity (Table 2).

**Table 2:** Alpha-diversity of soil microbiomes (data are expressed as mean ± S.E.M.)

	Index			
Soil	Shannon	Chao1	Number of OTUs	Faith PD
**Archived sod-podzolic (ASp)**	**5.19 ± 0.53**	**280.04 ± 36.96**	**142.70 ± 17.14**	**17.28 ± 3.12**
**Archived chernozem (ACh)**	**6.18 ± 0.35**	**466.57 ± 78.79**	**206.81 ± 26.23**	**19.18 ± 2.98**
**Modern Sod-podzolic (Sp)**	6.99 ± 0.63	509.96 ± 113.11	268.91 ± 49.12	28.44 ± 3.32
**Modern Chernozem (Ch)**	6.77 ± 0.58	415.85 ± 54.55	243.01 ± 38.70	27.35 ± 3.37

According to the analysis of biodiversity indices, the phylogenetic diversity of both types of archived soils was significantly lower than of the modern ones. The observed species richness and entropy did not differ significantly between soils. However, the estimated number of species and Shannon index were significantly lower in samples of ASp soil.

### β-diversity analysis of soil microbiomes

**Fig 3:**
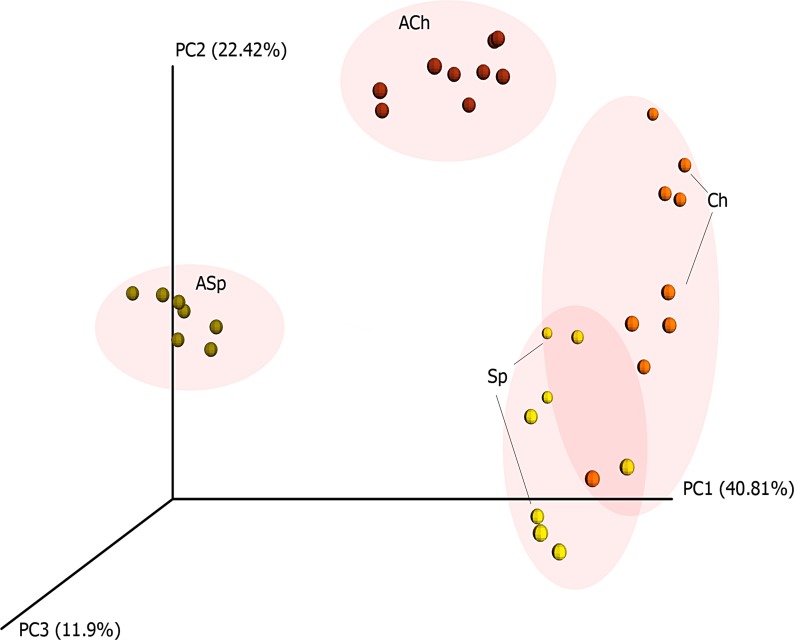
PCoA of weighted UniFrac distance matrices calculated for soil microbiomes. ACh–samples of Archived Chernozem soil, ASp–samples of Archived Sod-podzolic soil, Sp and Ch–samples of modern Sod-podzolic soil and Chernozem respectively.

The weighted UniFrac distance matrices were used to reveal if the long-term preservation had a significant effect on the microbial complexes of investigated soils. The Principal Coordinates Analysis (PCoA) analysis of the weighted UniFrac distance matrices showed that samples of archived soils generally formed clear separated clusters, whereas the distance between clusters of modern sod-podzolic (Sp) and chernozem (Ch) soils was relatively smaller (Fig 3). This finding was supported by both anosim and adonis ordination models (p < 0.0001). At the same time, the distance between clusters of ACh and (Sp+Ch) was less than the distance between these clusters and the ASp samples cluster. Indeed, adonis showed that chernozems better preserve the structure of the modern communities. This is also consistent with the lower values of conserved biodiversity in the ASp soils in comparison with ACh and modern soils (Table 2). The adonis model showed that sample type and sample age explained the same portion of variation in the data.

Thus, the analysis of biodiversity revealed the substantial loss of **archaeal and bacterial** diversity in both types of archived soil samples with most dramatic changes in ASp soils.

### Taxonomic analysis of microbiomes of archived and modern soils

Most of soil samples were dominated by 8 bacterial and one archaeal phyla, they were: *Actinobacteria* (27.4%), *Firmicutes* (23.2%), *Proteobacteria* (17.4%), *Verrucomicrobia* (9.1%), *Acidobacteria* (6.6%), *Bacteroidetes* (3.8%), *Chloroflexi* (2.4%), *Gemmatimonadetes* (1.2%) and *Crenarchaeota* (6.5%). Rare phylotypes (less than 1% in amount) included *Planctomycetes* (0.6%), AD3 (0.1%), *Nitrospirae* (0.1%), WS3 (0.1%) and *Euryarchaeota* (0.2%).

**Fig 4:**
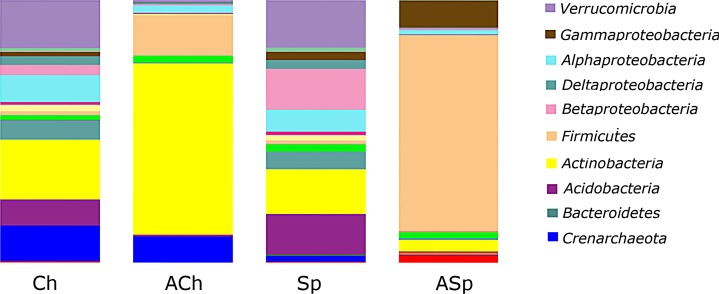
Taxa summary of microbiomes of soils investigated at the phylum level, including classes of *Proteobacteria*.

At the phylum level, strong differences were found between microbial communities of archived soils and modern soils, as well as the abundance of some phylotypes differed markedly between ACh and ASp microbiomes (Fig 4). Archived soils were characterized by relative increasing in amount of *Firmicutes* (especially ASp soils) and substantial decreasing in number of *Proteobacteria*, *Verrucomicrobia*, *Acidobacteria*, *Bacteroidetes* (Fig 4). At the same time, ACh were dominated by actinobacteria and were characterized by preservation of Archaea (*Crenarchaeota* phylotypes) in comparison to ASp soil.

The microbiomes of archived soils and their modern counterparts differed markedly in the composition of phylum *Proteobacteria* (Fig 4). Both Ch and Sp soils were dominated by *Alphaproteobacteria* and *Betaproteobacteria*, while in ACh soil the proportion of *Alphaproteobacteria* decreases to 2.73% (in comparison with 10.19% and 17.31% in Ch and Sp, respectively), whereas in ASp soil proteobacteria were represented mainly by gammaproteobacteria (9.98% in comparison with 2.46% and 1.61% in Sp and Ch, respectively).

**Fig 5:**
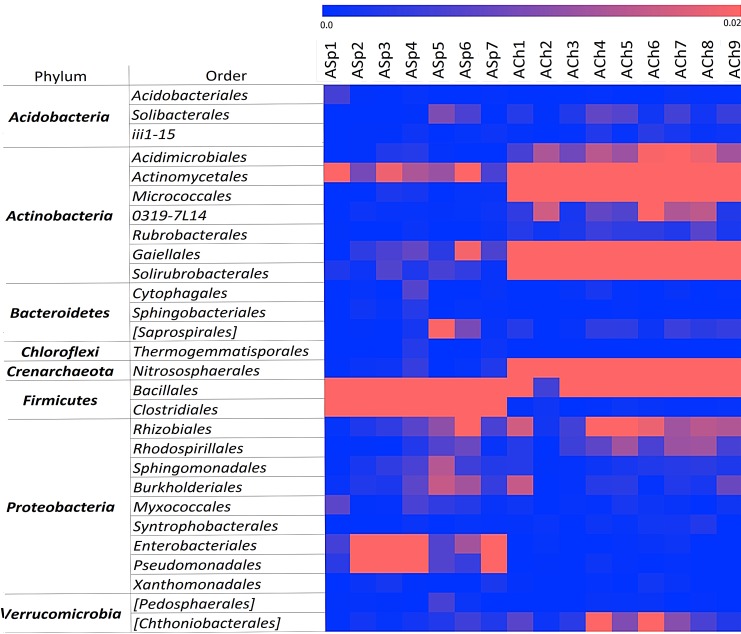
Heatmap of fractions of microbial orders* in archived sod-podzolic and chernozem soils. 0.01 units = 1% of the sample * doesn’t include the orders with relative abundance below 0.001 in the dataset of each soil type.

Fig 5 represents the differences in taxonomic composition of archived sod-podzolic and chernozem soils. The microbial community of ASp soil consisted mainly of spore-forming bacteria (orders *Bacillaceae* and *Clostridiaceae*) and some portion of actinobacteria of *Actinomycetales*, *Acidimicrobiales*, *Gaiellales*, *Solirubrobacterales* orders. These soils were also characterized by the increasing in abundance of gammaproteobacetria of *Entherobacterales* and *Pseudomonadales* and the presence of some other proteobacteria (*Rhizobiales*, *Burkholderiales*). Microbiomes of archived chernozems included mainly actinobacteria: thermophilic (bacteria from orders *Gaiellales*, *Solirubrobacterales*), mycelia-forming (*Actinomycetales*) and others (*Micrococcales*, *Rubrobacterales*). At the same time *Clostridia* were absent in these samples and *Firmicutes* were presented mainly by bacilli.

Phyla-level beta-regression relative abundance models, fitted for *Acidobacteria*, *Actinobacteria*, *Bacteroidetes*, *Chloroflexi*, *Crenarchaeota*, *Firmicutes*, *Gemmatimonadetes*, *Planctomycetes*, *Proteobacteria*, *Verrucomicrobia* in ACh and Ch samples, mostly supported significant predictable effects of long-term soil storage (R^2^ = 0.7–0.9). All phyla, except *Chloroflexi*, were significantly (p < 0.01) influenced by sample type. More importantly, only 2 phyla (*Acidobacteria* and *Verrucomicrobia*) were significantly (p < 0.02) affected by the difference in vegetation type. At the same time, *Acidobacteria*, *Bacteroidetes*, *Gemmatimonadetes* and *Verrucomicrobia* demonstrated significant (p < 0.05) response to the interaction between sample type and vegetation type.

**Fig 6:**
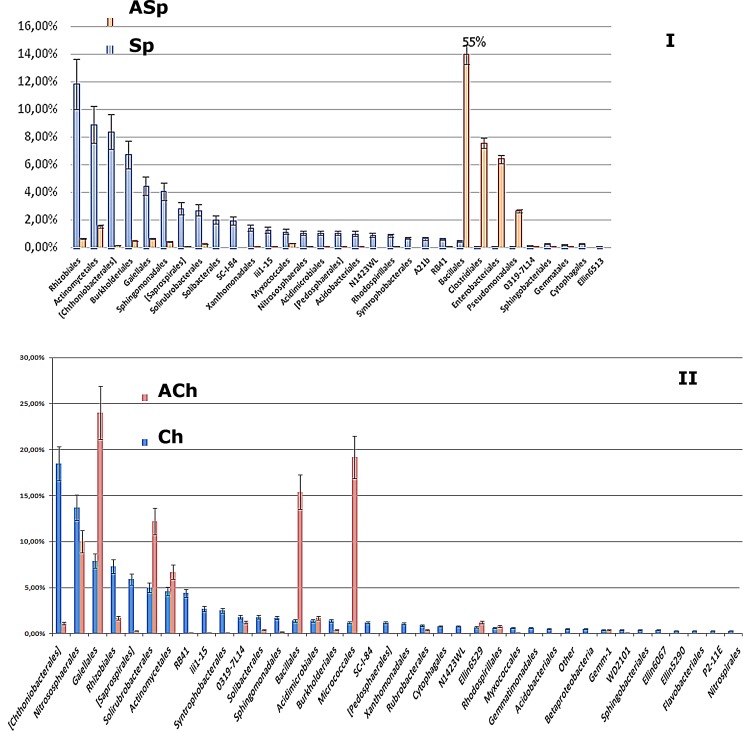
Comparative analysis of taxonomic structure of microbiomes* of archived and modern soils. The charts were constructed by ranging microbial taxa abundances based on relative taxa proportions in modern soils. 1 unit = 1% of the corresponding dataset. * doesn’t include the orders with relative abundance below 0.6% in the total dataset

The comparison of ASp and ACh soils with their modern counterparts revealed that long-term storage of sod-podzolic soil leads to the significant loss of its biodiversity and the increase in the relative abundance of minor taxa (Fig 6). However, in ACh relatively diverse microbial communities are preserved with the dominance of major actinobacterial taxa, which also prevail in Ch soils.

## Discussion

The study demonstrates that despite the air-drying and long-term storage, archived soils can retain specific microbial communities resembling that of their modern counterparts. However, the structure and composition of the microbiome, and the level of genetic material preservation depend largely on the physicochemical properties that differ across soil types. It is shown that the preservation of genetic material in terms of both quantity and composition was significantly higher in chernozem samples in comparison with archived samples of sod-podzolic soils. Chernozem samples contained high concentrations of nondegraded DNA, while most of the DNA in archived sod-podzolic samples was degraded. This leads us to conclude that the analysis of archived microbiomes is more valuable in case of soils with high humus content with good water-physical and agro-ecological characteristics, such as chernozems.

Although community separation tests support these observations, some important test assumptions were not met. Anosim and adonis are not robust to category imbalance in the experimental design. More importantly the lack of proper randomization across factor interactions in the experimental design results in the violation of multivariate spread among groups, which can ultimately lead to false positive results. It is also possible that archive chernozem samples had higher initial microbial content, which, assuming constant DNA-degradation rate, would’ve resulted in higher DNA-yields. At the same time, our adonis model, incorporating sample type and sample age, showed that the latter factor explained no additional variation, which allowed us to assume nonlinear DNA-degradation rate with fast slowdown.

The study of microbiomes of archived samples of chernozem and sod-podzolic soils revealed the dominance of components that are capable of long-term survival under the unfavorable ecological conditions due to the formation of endospores (*Bacillus*, *Clostridium*), as well as thermotolerant microorganisms (*Actinobacteria*), that are often a part of the microbiomes of dry ecotopes. Although the prolonged air-drying of soils to a certain extent leads to the loss of viability and cellular integrity with concomitant loss of genetic material of soil microbiomes [27, 28], some microorganisms can produce dormant forms—spores and cysts—and thus can withstand adverse environmental conditions. The relative abundance of *Actinobacteria* in soils with low values of humidity is due to the biological characteristics of this bacterial group that ensure their existence in such extreme environmental conditions [29]. Since we did not explicitly account for humidity variation (prior and during storage) in our models we couldn’t completely strike out the effects they had on the observed differences in the abundance of *Actinobacteria*.

The data obtained are consistent with the results of the analysis of archived soil samples of the Rothamsted Experimental Station [16]. The authors emphasized the preservation of *Firmicutes* in the archived soil samples, mainly g. *Bacillus*, as well as a significant loss in the diversity of archived soils in comparison to the modern ones. However, in this study the microbiome was analyzed by DGGE method–the technique with relatively low resolution that targets only the most dominant bacterial taxa, while samples under investigation were of the same soil type and differed only in the type of fertilizers application [16].

Here we used high-throughput sequencing of 16 S rRNA gene to investigate the microbial community of archived samples of two considerably different genetic soil types (sod-podzolic soils and chernozems), which was never done before. The investigated soils are formed in different natural zones with contrasting characteristics of moisture and temperature, and thus differ significantly in their physical and chemical properties. Chernozems that emerge in a warmer climate under steppe vegetation demonstrate high levels of organic matter, nitrogen and biophilic elements content, as well as better aggregation compared to the sod-podzolic soils. The latter are formed mainly on light-textured parent material in conditions of a percolated water regime, so the intensity of humification process in these soils is weakened, leading to the worse water-physical properties and loss of soil organic matter (SOM) and biophilic elements content [30, 31, 32].

The recent research studies demonstrate the differences in microbial communities of archived soil samples with different content and quality of SOM [33]. Soils with a high content of organic matter (due to the long-term application of manure) were characterized by higher microbial abundance than soils with long-term use of mineral fertilizers. The microbial activity decreased more sharply over time after rewetting in case of mineral fertilizers application (with a lower content of SOM) in comparison to the samples with organic farming system [33]. It seems that the high content of organic matter coupled with good aggregation of chernozems lead to greater levels of preservation of the microbial DNA in these soils, and therefore, contributes to the maintenance of a relatively high biodiversity in the archived chernozem in comparison to sod-podzolic soil. The samples of archived sod-podzolic soils were characterized by minimal values of biodiversity, the relatively smaller amount of total DNA and 16 S rRNA ribosomal operons in comparison to the archived chernozem and samples of modern sod-podzolic soil and chernozem.

From a fundamental point of view, the development of the future research in this direction is of paramount importance for the study of soil genesis and the evolution of the soil microbiome. The detection of bacilli, many of which are part of the associative community of PGP (plant grow promoting) bacteria, and *Actinobacteria* (bacteria of *Actinomycetales* order), most of which are producers of antibiotics and growth-stimulating agents, in the microbial community of archived soil samples makes studying these soils very promising for agricultural microbiology. Samples of archived soils can serve as a source for the isolation of associative and symbiotic microorganisms, which is promising for the creation of new agricultural microbial preparations, as well as specific ancestral genetic constructs, which have not experienced the degradative evolutionary transformations caused by anthropogenic factor.

## Supporting information

S1 Fig Electropherogram of DNA in agarose gele (uncropped). М–marker of λ phage DNA treated by Hindt 3 restrictase, ACh–Samples of Archived Chernozem (ACh), Ch–Modern (Control) Chernozem (Ch), ASp–Samples of Archived Sod-podzolic soil (ASp), Sp–Modern (Control) Sod-podzolic (Sp) soil.
(TIF)
