# Supplementary figures and images for: The preservation of microbial DNA in archived soils of various genetic types
Source: PLoS One. 2017 Mar 24;12(3):e0173901. doi: 10.1371/journal.pone.0173901 (PMC5365134; doi:10.1371/journal.pone.0173901)

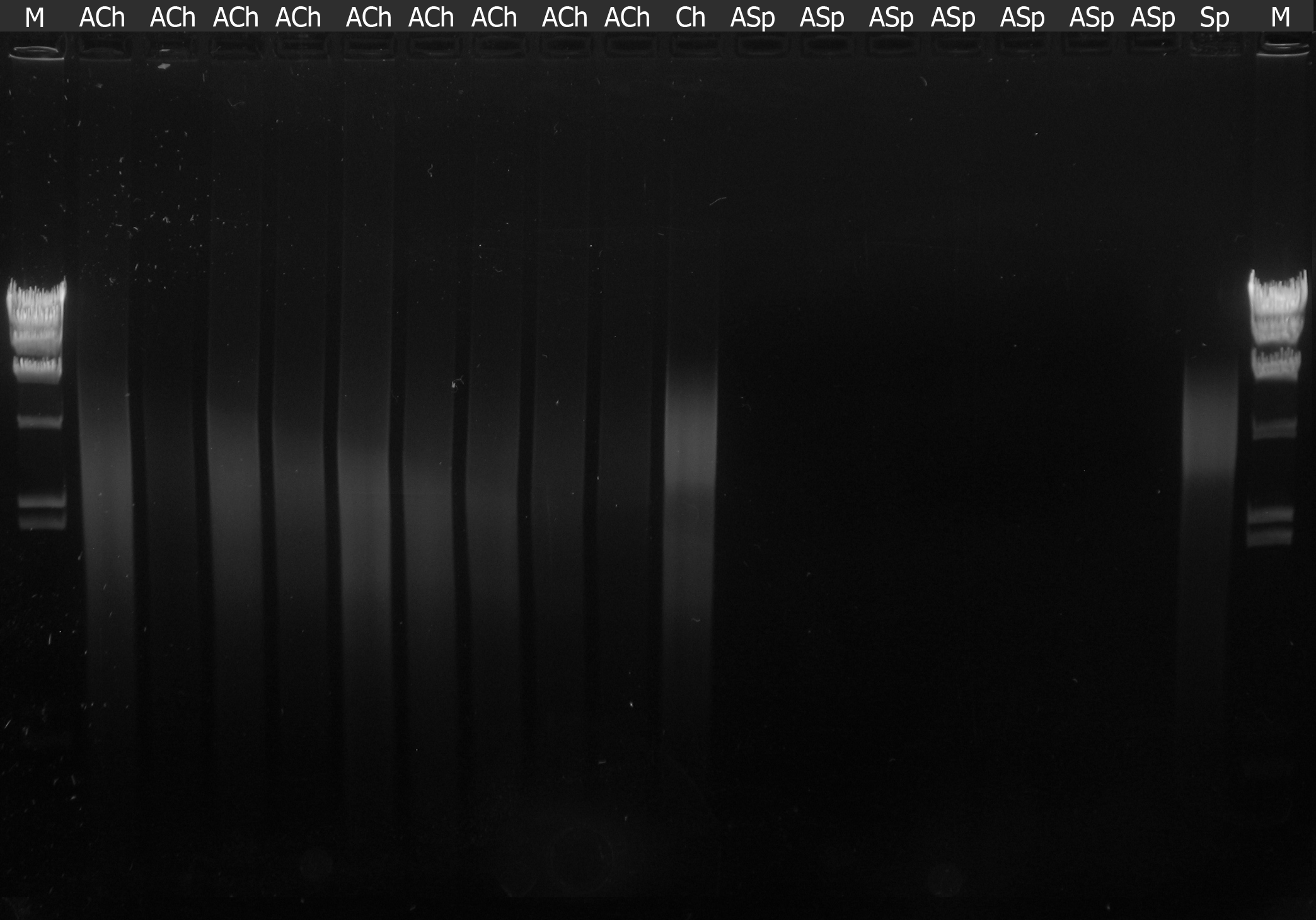

Supplement: S1 Fig — М–marker of λ phage DNA treated by Hindt 3 restrictase, ACh–Samples of Archived Chernozem (ACh), Ch–Modern (Control) Chernozem (Ch), ASp–Samples of Archived Sod-podzolic soil (ASp), Sp–Modern (Control) Sod-podzolic (Sp) soil. (TIF) [file pone.0173901.s001.tif]
